# Transcriptomic analysis of differentially expressed genes in leaves and roots of two alfalfa (*Medicago sativa* L.) cultivars with different salt tolerance

**DOI:** 10.1186/s12870-021-03201-4

**Published:** 2021-10-05

**Authors:** Surendra Bhattarai, Yong-Bi Fu, Bruce Coulman, Karen Tanino, Chithra Karunakaran, Bill Biligetu

**Affiliations:** 1 Department of Plant Sciences, College of Agriculture and Bioresources, University of Saskatchewan, 51 Campus Drive, Saskatoon, SK S7N 5A8 Canada; 2 Plant Gene Resources of Canada, Saskatoon Research and Development Centre, Agriculture and Agri-Food Canada, 107 Science Place, Saskatoon, SK S7N 0X2 Canada; 3 Canadian Light Source, 44 Innovation Boulevard, Saskatoon, SK S7N 2V3 Canada

**Keywords:** Alfalfa, Differentially expressed genes, Salt stress, Transcriptome

## Abstract

**Background:**

Alfalfa (*Medicago sativa* L.) production decreases under salt stress. Identification of genes associated with salt tolerance in alfalfa is essential for the development of molecular markers used for breeding and genetic improvement.

**Result:**

An RNA-Seq technique was applied to identify the differentially expressed genes (DEGs) associated with salt stress in two alfalfa cultivars: salt tolerant ‘Halo’ and salt intolerant ‘Vernal’. Leaf and root tissues were sampled for RNA extraction at 0 h, 3 h, and 27 h under 12 dS m^− 1^ salt stress maintained by NaCl. The sequencing generated a total of 381 million clean sequence reads and 84.8% were mapped on to the alfalfa reference genome. A total of 237 DEGs were identified in leaves and 295 DEGs in roots of the two alfalfa cultivars. In leaf tissue, the two cultivars had a similar number of DEGs at 3 h and 27 h of salt stress, with 31 and 49 DEGs for ‘Halo’, 34 and 50 for ‘Vernal’, respectively. In root tissue, ‘Halo’ maintained 55 and 56 DEGs at 3 h and 27 h, respectively, while the number of DEGs decreased from 42 to 10 for ‘Vernal’. This differential expression pattern highlights different genetic responses of the two cultivars to salt stress at different time points. Interestingly, 28 (leaf) and 31 (root) salt responsive candidate genes were highly expressed in ‘Halo’ compared to ‘Vernal’ under salt stress, of which 13 candidate genes were common for leaf and root tissues. About 60% of DEGs were assigned to known gene ontology (GO) categories. The genes were involved in transmembrane protein function, photosynthesis, carbohydrate metabolism, defense against oxidative damage, cell wall modification and protection against lipid peroxidation. Ion binding was found to be a key molecular activity for salt tolerance in alfalfa under salt stress.

**Conclusion:**

The identified DEGs are significant for understanding the genetic basis of salt tolerance in alfalfa. The generated genomic information is useful for molecular marker development for alfalfa genetic improvement for salt tolerance.

**Supplementary Information:**

The online version contains supplementary material available at 10.1186/s12870-021-03201-4.

## Background

Alfalfa (*Medicago sativa* L.) is an important forage legume in the world. Cultivated alfalfa is an outcrossing autotetraploid (2n = 4x = 32) with a genome size of 800–1000 Mb [1]. Although alfalfa is regarded as moderately tolerant to salinity [2], alfalfa yield reduces by approximately 6–7% for each dS m^− 1^ increase above a salinity of 2 dS m^− 1^ [3]. To stabilize alfalfa production under saline regions, the development of superior salt tolerant cultivars becomes an important breeding goal. Identification of candidate genes for salt tolerance can increase the accuracy of parental selection as this trait has low heritability [4]. Salt tolerance is a complex trait controlled by multiple genes, involving different signaling pathways, osmotic tolerance, ion transport, compartmentalization of salt ions in vacuoles, the synthesis of plant hormones and photosynthesis [5].

Next-generation sequencing technologies have been used to identify candidate genes involved in salt tolerance of alfalfa. Transcriptomic studies in the 1-week old root tissue of alfalfa under salt stress found 1165 DEGs, including 86 transcription factors, which are responsible for stress tolerance, kinase, hydrolase, and oxidoreductase activities [6]. Luo et al. [7] identified 8861 DEGs in 12-day old seedlings of alfalfa under salt stress, which are responsible for ion homeostasis, antiporter, signal perception, signal transduction, transcriptional regulation, and antioxidative defense. Lei et al. [8] revealed 2237 DEGs between salt tolerant and intolerant alfalfa cultivars and found a salt tolerant alfalfa cultivar maintained relatively stable expression of genes responsible for reactive oxygen species and Ca^2+^ pathway, phytohormone biosynthesis and Na^+^/K^+^ transport under stress. Gruber et al. [9], using bulked genotypes as replications, studied transcriptomes in alfalfa and found genes responsible for numerous functions in a salt intolerant alfalfa cultivar. In recent years, genetic modification of certain genes controlling salt tolerance have also been conducted in alfalfa. Overexpression of salt responsive genes or transcription factors had improved salt tolerance in transgenic alfalfa. Such genes include *Alfin1* [10], *AVP1* [11], *GmDREB1* [12], *SsNHX1* [13], *TaNHX2* [14], *GsCBRLK* [15], *GsZFP1* [16], *OsAPX2* [17], *SeNHX1* [18], *AtNDPK2* [19], *AgcodA* [20], and *GsWRKY20* [21]. These studies have advanced our understanding of the genetic control for salt tolerance in alfalfa. However, most studies mainly focused on single time point sampling of root tissue at the seedling stage after salt stress, limiting the analysis of the temporal expression of genes affecting salt tolerance.

Tissue specific protein induction is regulated during salinity stress and is unique to roots and shoots [22]. Thus, there should be tissue specific transcriptomic responses [23–25]. Although the root is the first receptor of salt stress [6, 7], leaf tissue is the main energy source for plant growth and stress tolerance during active growth and developmental stages. To advance our knowledge about the temporal gene expression in different tissues for the genetic control under salt stress between tolerant and intolerant cultivars, we conducted a RNA-Seq analysis with the objective to simultaneously analyze gene expressions of leaf and root tissues of two alfalfa cultivars with different tolerance to salinity after exposing them to 12 dS m^− 1^ of electrical conductivity salt stress for 0 h, 3 h, and 27 h. The analysis was fruitful with the identification of many unique genes conditioning salt tolerance in alfalfa.

## Results

### High throughput sequencing and assembly

A total of 408 million raw sequence reads were generated using the Illumina HiSeq sequencing platform. The reads were reduced to 93.5% (381 million clean reads) by removing adapter contamination and reads with length lower than 36 bp (Table 1). There were 84.8% of clean reads mapped to the alfalfa reference genome using STAR (v2.6.1a). The samples showed high percentages (78.8–92.4%) of mapping with the alfalfa reference genome except for ‘Halo’ root tissue sampled at 27 h of salt stress.

**Table 1:** Summary of Illumina sequencing data and mapped sequence reads for the assayed alfalfa samples

Genotype	Tissue	Treatment	Biological replicate	Total reads	Mapped reads	Mapping rate (%)
Halo	Leaf	Control	1	7,235,661	6,275,026	86.7
		Control	2	7,462,991	6,607,722	88.5
		Control	3	7,532,603	6,371,474	84.6
		Control	4	7,163,647	6,286,906	87.8
		Stressed (3h)	1	7,170,087	6,291,489	87.7
		Stressed (3h)	2	8,619,083	7,531,679	87.4
		Stressed (3h)	3	7,275,223	6,453,594	88.7
		Stressed (3h)	4	7,346,150	6,417,574	87.4
		Stressed (27h)	1	7,234,036	5,968,374	82.5
		Stressed (27h)	2	7,186,154	6,225,839	86.6
		Stressed (27h)	3	6,256,894	5,471,745	87.5
		Stressed (27h)	4	5,696,229	4,741,642	83.2
	Root	Control	1	7,930,008	6,833,311	86.2
		Control	2	7,568,654	6,104,814	80.7
		Control	3	10,017,590	9,054,027	90.4
		Control	4	6,523,142	5,561,719	85.3
		Stressed (3h)	1	9,003,316	7,662,274	85.1
		Stressed (3h)	2	11,023,879	9,201,753	83.5
		Stressed (3h)	3	9,647,653	8,529,106	88.4
		Stressed (3h)	4	6,201,499	4,884,633	78.8
		Stressed (27h)	1	6,563,910	4,983,668	75.9
		Stressed (27h)	2	8,321,924	3,727,203	44.8
		Stressed (27h)	3	9,424,651	7,313,948	77.6
		Stressed (27h)	4	7,942,407	2,854,933	35.9
Vernal	Leaf	Control	1	7,085,736	6,147,440	86.8
		Control	2	7,149,929	6,449,664	90.2
		Control	3	8,108,009	7,362,827	90.8
		Control	4	10,775,421	9,953,409	92.4
		Stressed (3h)	1	5,372,062	4,901,710	91.2
		Stressed (3h)	2	6,180,723	5,477,930	88.6
		Stressed (3h)	3	7,355,464	6,390,995	86.9
		Stressed (3h)	4	6,775,443	6,098,490	90.0
		Stressed (27h)	1	11,398,645	10,388,765	91.1
		Stressed (27h)	2	7,517,258	6,754,908	89.9
		Stressed (27h)	3	8,128,644	7,280,110	89.6
		Stressed (27h)	4	7,713,476	7,007,553	90.8
	Root	Control	1	11,004,685	9,744,748	88.6
		Control	2	10,402,513	9,091,041	87.4
		Control	3	8,070,744	7,001,864	86.8
		Control	4	8,936,244	7,616,107	85.2
		Stressed (3h)	1	8,293,827	7,159,751	86.3
		Stressed (3h)	2	8,722,568	7,705,076	88.3
		Stressed (3h)	3	9,769,559	8,854,805	90.6
		Stressed (3h)	4	6,402,547	5,317,429	83.1
		Stressed (27h)	1	3,669,213	3,121,348	85.1
		Stressed (27h)	2	9,000,614	7,858,878	87.3
		Stressed (27h)	3	8,661,229	7,569,795	87.4
		Stressed (27h)	4	8,640,454	7,481,651	86.6
Total				381,482,398	324,090,747	
Average (Control)				8,310,474	7,278,881	87.4
Average (3 h)				7,882,443	6,804,893	87.0
Average (27 h)				7,709,734	6,171,898	80.1
Average				7,947,550	6,751,891	84.8

### Differentially expressed genes (DEGs)

**Fig. 1:**
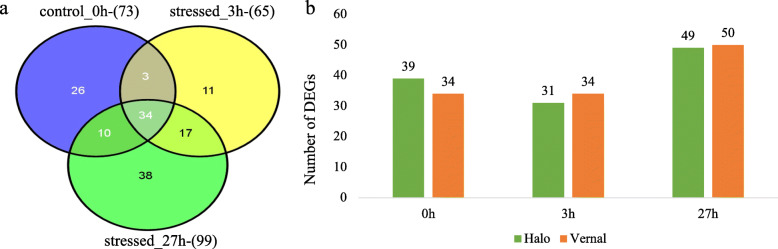
Differentially expressed genes (DEGs) between salt tolerant ‘Halo’ and intolerant ‘Vernal’ alfalfa cultivars in leaf tissue at three different time-points: control (0 h), 3 h and 27 h after salt stress. **a** Venn diagram for number of DEGs in leaf tissue of two alfalfa cultivars (‘Halo’ vs. ‘Vernal’) at three different time-points (0 h, 3 h, and 27 h). Number in the parenthesis represents total number of DEGs. Numbers in each intersection represent the number of DEGs detected in two or three time points. **b** Number of DEGs identified in leaf tissue at each time-point (0 h, 3 h, and 27 h) between tolerant and intolerant alfalfa cultivars

In leaf tissue, there were 237 DEGs identified between the two alfalfa cultivars. Among them, 34 DEGs were expressed at all three time points (0 h, 3 h, and 27 h) and 17 DEGs expressed after exposing to salt stress (3 h and 27 h) (Fig. 1a, b; Additional file 1: Table S1). Of these DEGs, 39, 31, and 49 DEGs were specific to ‘Halo’, and 34, 34, and 50 DEGs were specific to ‘Vernal’ at 0 h, 3 h and 27 h of salt stress, respectively (Fig. 1b). The number of DEGs in leaf tissue decreased after 3 h compared to 0 h treatment. Then, the number of DEGs increased from 3 h to 27 h of salt stress for both cultivars (Fig. 1b).

**Fig. 2:**
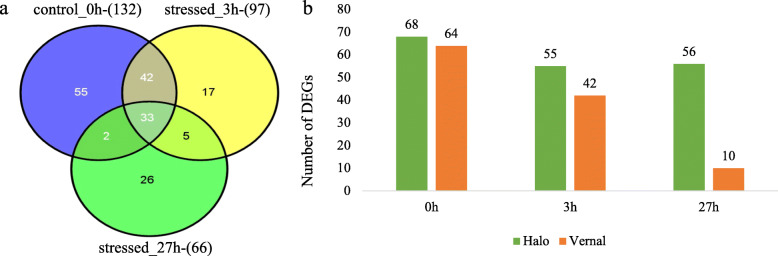
Differentially expressed genes (DEGs) between salt tolerant ‘Halo’ and intolerant ‘Vernal’ alfalfa cultivars in root tissue at three different time-points: control (0 h), 3 h and 27 h after salt stress. **a** Venn diagram for number of DEGs in root tissue of two alfalfa cultivars (‘Halo’ vs. ‘Vernal’) at three different time-points (0 h, 3 h, and 27 h). Number in the parenthesis represents total number of DEGs. Numbers in each intersection represent the number of DEGs detected in two or three time points. **b** number of DEGs identified in root tissue at each time-point (0 h, 3 h, 27 h) between tolerant and intolerant alfalfa cultivars

In root tissue, a total of 295 DEGs were identified between the two alfalfa cultivars. There were 33 DEGs expressed at all three time points and 5 DEGs expressed after exposing to salt stress (Fig. 2a, b; Additional file 1: Table S2). Of these DEGs, 68, 55, and 56 DEGs were specific to ‘Halo’ at 0 h, 3 h and 27 h, whereas 64, 42, and 10 DEGs were specific to ‘Vernal’, respectively (Fig. 2b). The number of DEGs in root tissue decreased at 3 h as compared to 0 h treatment for both cultivars, but the decrease was greater for ‘Vernal’ than for ‘Halo’. The main difference of DEGs between the two cultivars in the root was from 3 h to 27 h, with a 76% decrease in DEGs in ‘Vernal’ while there was almost no change for ‘Halo’ (Fig. 2b). After 27 h of salt stress in root tissue, the number of DEGs in ‘Halo’ were five times more than that of ‘Vernal’ (Fig. 2b).

### Functional annotation of DEGs

**Fig. 3:**
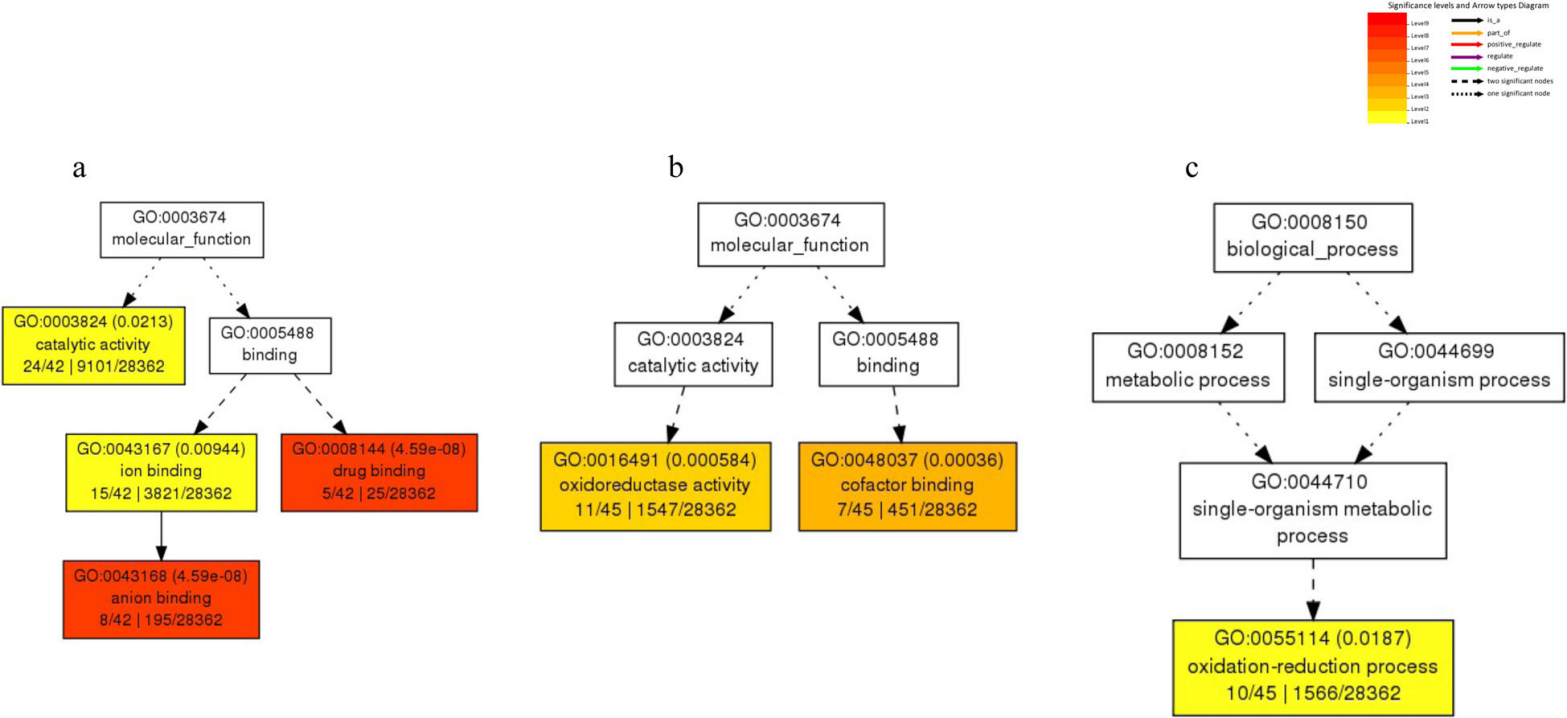
Gene ontology (GO) analysis of differentially expressed genes in leaf tissues of two alfalfa cultivars in response to salt stress (**a**, salt tolerant ‘Halo’ alfalfa; **b** and **c**, salt intolerant ‘Vernal’ alfalfa). Each box shows the GO term number, *p*-value in parenthesis, and GO term

To understand what biological processes are implicated in response to salinity, we assigned the DEGs to known Gene Ontology (GO) categories. Among 237 DEGs in leaf tissue, 148 (62.4%) DEGs were assigned to three ontology classes. In ‘Halo’ leaf tissue, the most noticeable DEGs [false discovery rate (FDR) < 0.05] were “drug binding” (GO:0008144, 5), “anion binding” (GO:0043168, 8), “ion binding” (GO:0043167, 15) and “catalytic activity” (GO:0003824, 24) among molecular functions (Fig. 3a) while there was no significantly enriched functional groups from biological process and cellular component. For ‘Vernal’ leaf tissue, “cofactor binding” (GO:0048037, 7) and “oxidoreductase activity” (GO:0016491, 11) were predominant (FDR < 0.05) among molecular functions (Fig. 3b) and “oxidation-reduction process” (GO:0055114, 10) (Fig. 3c) in biological process, but there was not any significantly enriched functional groups from cellular component.

**Fig. 4:**
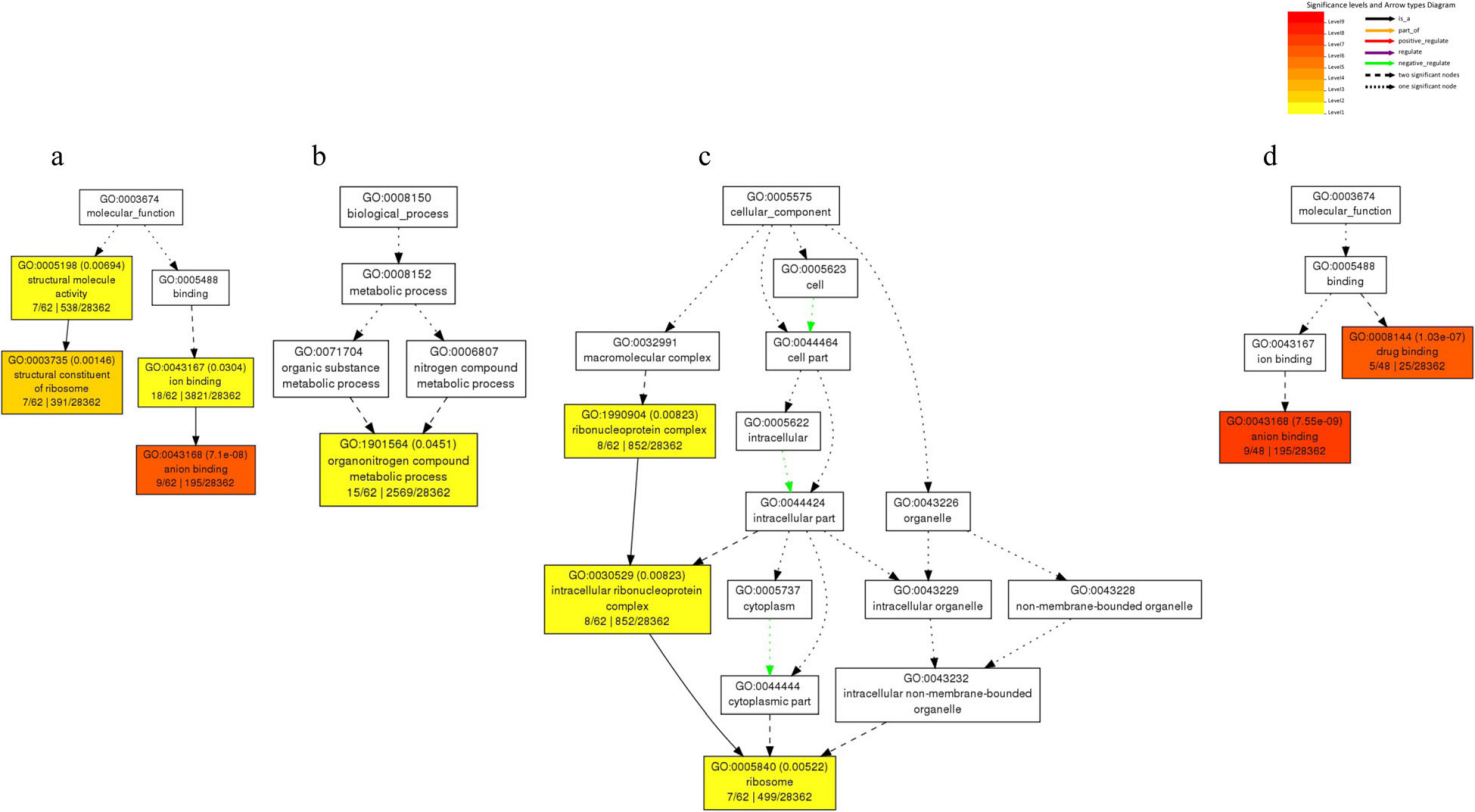
Gene ontology (GO) analysis of differentially expressed genes in leaf tissues of two alfalfa cultivars in response to salt stress (**a**, salt tolerant ‘Halo’ alfalfa; **b** and **c**, salt intolerant ‘Vernal’ alfalfa). Each box shows the GO term number, *p*-value in parenthesis, and GO term

Among the 295 DEGs in root tissue, 180 (61.0%) DEGs were annotated to three gene ontology classes. In root tissue of ‘Halo’, “anion binding” (GO:0043168, 9), “ion binding” (GO:0043167, 18) , “structural constituent of ribosome” (GO:0003735, 7), and “structural molecule activity” (GO:0005198, 7) among molecular functions (Fig. 4a) were noticeable, while “organo-nitrogen compound metabolic process” (GO:1901564, 15) was dominant among biological processes (Fig. 4b). “Ribosome” (GO:0005840, 7), “ribonucleoprotein complex” (GO:1990904, 8), “intracellular ribonucleoprotein complex” (GO:0030529, 8) were predominant in cellular components (Fig. 4c). For root tissue of ‘Vernal’, “anion binding” (GO:0043168, 9) and “drug binding” (GO:0008144, 5) (Fig. 4d) were significantly (FDR < 0.05) enriched, while no other functional group from biological processes and cellular components.

To identify pathways involved in salt tolerance, we carried out Kyoto Encyclopedia of Genes and Genomes (KEGG) pathways analysis of the DEGs. In total, 64 (27%) DEGs from leaf tissue and 86 (29.15%) DEGs from root tissue were assigned to 65 KEGG pathways (Table 2). In both tissues, the most significant DEGs were represented in the pathways of metabolism and biosynthesis of secondary metabolites. Of these, five pathways were common among different time points and alfalfa tissues. The highest level of enriched DEGs were in 14 pathways in leaf tissue and 6 pathways in root tissue after 27 h of salt stress. Among these pathways, the three highest enriched DEGs were involved in plant hormone signal transduction.

**Table 2:** Number of differentially expressed genes (DEGs) and corresponding pathways in leaf and root of salt tolerant ‘Halo’ and salt intolerant ‘Vernal’ alfalfa cultivars at 0, 3 and 27 h of salt stress

Pathway ID	The number of differentially expressed genes						Pathway terms
	HL0vsVL0	HL3vsVL3	HL27vsVL27	HR0vsVR0	HR3vsVR3	HR27vsVR27	
K00130	0	0	0	1	1	1	betB, gbsA; betaine-aldehyde dehydrogenase
K00276	0	0	1	0	0	0	AOC3, AOC2, tynA; primary-amine oxidase
K00430	0	0	1	0	0	0	E1.11.1.7; peroxidase
K00454	0	0	1	0	0	0	LOX2S; lipoxygenase
K00522	0	0	1	0	0	0	FTH1; ferritin heavy chain
K00549	1	1	1	1	1	1	metE; 5-methyltetrahydropteroyltriglutamate--homocysteine methyltransferase
K00660	0	0	0	0	0	1	CHS; chalcone synthase
K00915	0	0	0	1	0	0	IPMK, IPK2; inositol-polyphosphate multikinase
K01507	0	0	0	1	1	1	ppa; inorganic pyrophosphatase
K01535	0	0	1	1	0	0	PMA1, PMA2; H+-transporting ATPase
K01623	0	0	0	1	1	0	ALDO; fructose-bisphosphate aldolase, class I
K01823	1	1	1	1	1	0	idi, IDI; isopentenyl-diphosphate Delta-isomerase
K01859	0	0	0	0	0	1	E5.5.1.6; chalcone isomerase
K02639	1	1	2	1	1	0	petF; ferredoxin
K02721	1	0	1	0	0	0	psbW; photosystem II PsbW protein
K02893	1	0	0	1	2	1	RP-L23Ae, RPL23A; large subunit ribosomal protein L23Ae
K02906	0	0	1	0	0	0	RP-L3, MRPL3, rplC; large subunit ribosomal protein L3
K02925	0	0	1	0	0	0	RP-L3e, RPL3; large subunit ribosomal protein L3e
K02971	0	0	1	1	1	0	RP-S21e, RPS21; small subunit ribosomal protein S21e
K02981	0	0	0	1	0	0	RP-S2e, RPS2; small subunit ribosomal protein S2e
K02985	1	0	0	0	0	0	RP-S3e, RPS3; small subunit ribosomal protein S3e
K02991	0	0	0	1	1	0	RP-S6e, RPS6; small subunit ribosomal protein S6e
K03231	2	2	2	2	2	2	EEF1A; elongation factor 1-alpha
K03283	0	0	0	0	0	1	HSPA1s; heat shock 70kDa protein 1/2/6/8
K03364	0	0	1	0	0	0	CDH1; cell division cycle 20-like protein 1, cofactor of APC complex
K05546	0	0	0	1	1	0	GANAB; mannosyl-oligosaccharide alpha-1,3-glucosidase
K06617	0	0	0	0	1	0	E2.4.1.82; raffinose synthase
K07374	0	0	0	1	0	0	TUBA; tubulin alpha
K07466	1	1	1	1	1	0	RFA1, RPA1, rpa; replication factor A1
K08678	0	0	0	1	0	0	UXS1, uxs; UDP-glucuronate decarboxylase
K09495	1	1	1	1	1	1	CCT3, TRIC5; T-complex protein 1 subunit gamma
K09588	0	1	0	0	0	0	CYP90A1, CPD; cytochrome P450 family 90 subfamily A1
K09645	0	0	0	1	0	0	CPVL; vitellogenic carboxypeptidase-like protein
K10534	0	0	0	0	1	0	NR; nitrate reductase (NAD(P)H)
K10573	0	0	0	0	0	1	UBE2A, UBC2, RAD6A; ubiquitin-conjugating enzyme E2 A
K10767	0	0	0	1	0	0	ALKBH5; mRNA N6-methyladenine demethylase
K11717	0	0	0	0	0	1	sufS; cysteine desulfurase / selenocysteine lyase
K12130	0	1	1	0	0	0	PRR5; pseudo-response regulator 5
K12236	1	1	1	1	1	1	NFX1; transcriptional repressor NF-X1
K12741	0	0	0	0	1	0	HNRNPA1_3; heterogeneous nuclear ribonucleoprotein A1/A3
K12891	0	1	0	0	0	0	SFRS2; splicing factor, arginine/serine-rich 2
K13946	0	0	1	1	1	0	AUX1, LAX; auxin influx carrier (AUX1 LAX family)
K13963	0	0	0	1	1	0	SERPINB; serpin B
K14315	0	1	1	1	1	0	NDC1, TMEM48; nucleoporin NDC1
K14404	0	0	0	0	1	0	CPSF4, YTH1; cleavage and polyadenylation specificity factor subunit 4
K14488	0	0	0	0	1	0	SAUR; SAUR family protein
K14504	0	0	0	0	0	1	TCH4; xyloglucan:xyloglucosyl transferase TCH4
K14568	0	0	1	1	1	1	EMG1, NEP1; rRNA small subunit pseudouridine methyltransferase Nep1
K14842	0	0	0	1	0	0	NSA2; ribosome biogenesis protein NSA2
K15281	0	0	0	1	0	0	SLC35D; solute carrier family 35
K15378	0	1	0	0	1	0	SLC45A1_2_4; solute carrier family 45, member 1/2/4
K15397	1	0	0	0	0	0	KCS; 3-ketoacyl-CoA synthase
K15747	1	0	1	0	0	0	LUT5, CYP97A3; beta-ring hydroxylase
K16298	0	0	1	1	1	0	SCPL-IV; serine carboxypeptidase-like clade IV
K17525	1	0	1	0	0	0	CHID1; chitinase domain-containing protein 1
K17592	0	0	0	0	1	0	SACS; sacsin
K17679	0	0	0	1	0	0	MSS116; ATP-dependent RNA helicase MSS116, mitochondrial
K18270	0	0	0	1	0	0	RAB3GAP1; Rab3 GTPase-activating protein catalytic subunit
K18857	0	0	0	1	1	0	ADH1; alcohol dehydrogenase class-P
K20471	0	0	1	0	0	0	COPD, ARCN1, RET2; coatomer subunit delta
K20628	1	0	0	0	1	0	exlX; expansin
K20726	0	1	1	1	1	1	TMEM222; transmembrane protein 222
K21797	0	0	1	1	0	0	SAC1, SACM1L; phosphatidylinositol 4-phosphatase
K23050	1	1	1	1	1	0	PCBER1; phenylcoumaran benzylic ether reductase
K23570	1	1	1	1	1	1	EMC10; ER membrane protein complex subunit 10

*HL0* Halo leaf control, *VL0* Vernal leaf control, *HL3* Halo leaf after 3 h of salt stress, *VL3* Vernal leaf after 3 h of salt stress, *HL27* Halo leaf after 27 h of salt stress, *VL27* Vernal leaf after 27 h of salt stress, *HR0* Halo root control, *VR0* Vernal root control, *HR3* Halo root after 3 h of salt stress, *VR3* Vernal root after 3 h of salt stress, *HR27* Halo root after 27 h of salt stress, *VR27* Vernal root after 27 h of salt stress

### Candidate genes to enhance salt tolerance in alfalfa

The detected DEGs can be classified into two major groups for the candidate genes responsible for salt tolerance in alfalfa: 1) genes consistently expressed under short-term and long-term salt stress (3 h and 27 h) in ‘Halo’, and 2) the genes consistently expressed at all three time points in ‘Halo’. In the first group, there were 13 genes (11 in leaf; 2 in root) consistently expressed at both 3 h and 27 h of salt stress. While in the second group, there were 46 genes (17 in leaf, 29 in root) consistently expressed at all three time points. Thirteen candidate genes were highly expressed in both leaf and root tissues of ‘Halo’ as compared to ‘Vernal’, while 15 and 18 candidate genes revealed tissue specific expression in the leaf and root tissues of ‘Halo’, respectively (Tables 3, 4, and 5). Among the genes expressed in both tissues, MS.gene029203 (F-box/LRR-repeat protein 4) showed increasing expression with time in both leaf and root tissues of ‘Halo’, while MS.gene049294 (caffeic acid 3-O-methyltransferase) showed increasing expression with time in leaf tissue and MS.gene01091 (T-complex protein 1 subunit gamma) and MS.gene32989 (hypothetical protein TSUD_06780) showed increasing expression with time only in root tissue. Among the genes with leaf tissue specific expression, MS.gene029201 (replication protein A 70 kDa DNA-binding subunit C), MS.gene029206 (FAD synthetase 1, chloroplastic), and MS.gene24098 (thioredoxin-like protein CDSP32 chloroplastic-like) showed increasing expression with time. Among the genes with root tissue specific expression, MS.gene011517 (14 kDa proline-rich protein DC2.15) and MS.gene013923 (histone lysine N-methyltransferase, H3 lysine-9 specific SUVH1), had higher and consistent expression with time under salt stress.

**Table 3:** List of 13 salt responsive candidate genes simultaneously highly expressed in both leaf and root tissues of salt tolerant alfalfa cultivar ‘Halo’

Gene ID	Nr ID^a^	log_2_FC^b^(Leaf)			log_2_FC (Root)			Putative function
		0h	3h	27h	0h	3h	27h	
MS.gene01091	XP_003593572.2	6.7	7.3	6.9	8.4	9.4	10.3	T-complex protein 1 subunit gamma [*Medicago truncatula* (barrel medic)]
MS.gene013211	XP_003602730.1	7.3	5.9	6.7	9.9	8.9	7.5	ribonuclease TUDOR 1 [*Medicago truncatula* (barrel medic)]
MS.gene013222	XP_003602710.1	5.5	5.9	5.5	7.6	6.7	7.3	cleft lip and palate transmembrane protein 1 homolog [*Medicago truncatula* (barrel medic)]
MS.gene017955	XP_003625216.1	5.5	6.9	5.6	NA	9.5	7.7	40S ribosomal protein S20-2 [*Medicago truncatula* (barrel medic)]
MS.gene029200	PNY01153.1	8.3	7.5	7.5	7.4	6.3	7.7	replication factor A protein [*Trifolium pratense*]
MS.gene029202	XP_013470381.1	7.7	8.1	7.2	8.2	8.1	8.5	E3 ubiquitin-protein ligase CIP8 [*Medicago truncatula* (barrel medic)]
MS.gene029203	XP_013470380.1	NA	6.8	7.3	6.8	8.0	8.5	F-box/LRR-repeat protein 4 [*Medicago truncatula* (barrel medic)]
MS.gene049294	XP_003602595.1	4.0	4.1	5.3	6.3	5.4	4.3	caffeic acid 3-O-methyltransferase [*Medicago truncatula* (barrel medic)]
MS.gene32989	GAU34467.1	NA	6.8	6.4	7.0	7.1	7.6	hypothetical protein TSUD_06780 [*Trifolium subterraneum*]
MS.gene36780	KEH43749.1	9.4	8.4	8.8	10.4	11.3	8.2	elongation factor 1-alpha [*Medicago truncatula* (barrel medic)]
MS.gene36960	AET01475.1	8.6	8.5	8.8	9.8	9.8	8.8	elongation factor 1-alpha [*Medicago truncatula*]
MS.gene52595	XP_003624202.1	7.3	8.0	7.7	9.5	8.1	7.9	ER membrane protein complex subunit 10 [*Medicago truncatula* (barrel medic)]
MS.gene93979	XP_003619874.1	7.7	6.9	7.9	7.4	7.4	7.0	NF-X1-type zinc finger protein NFXL1 [*Medicago truncatula* (barrel medic)]

^a^Nr ID is the protein accession number in NCBI non redundant protein database

^b^log2FC stands for log Fold Change, where it is log base 2

**Table 4:** List of 15 salt responsive candidate genes highly expressed in leaf tissue of salt tolerant alfalfa cultivar ‘Halo’

Gene ID	Nr ID^a^	log_2_FC^b^ (Leaf)			Putative function
		0h	3h	27h	
MS.gene024018	KHN29288.1	NA	8.9	4.9	Monothiol glutaredoxin-S14, chloroplastic [*Glycine soja*]
MS.gene029055	AFK45194.1	5.2	7.3	5.8	CDP-diacylglycerol--glycerol-3-phosphate 3-phosphatidyltransferase 2 [*Medicago truncatula* (barrel medic)]
MS.gene029201	AET03044.2	NA	7.5	9.0	replication protein A 70 kDa DNA-binding subunit C [*Medicago truncatula* (barrel medic)]
MS.gene029206	XP_024628388.1	NA	4.7	5.0	FAD synthetase 1, chloroplastic [*Medicago truncatula* (barrel medic)]
MS.gene037960	XP_003589866.2	NA	2.7	2.7	nuclear pore complex protein NUP1 [*Medicago truncatula* (barrel medic)]
MS.gene038586	RHN67456.1	NA	6.5	6.2	putative minus-end-directed kinesin ATPase [*Medicago truncatula*]
MS.gene065734	XP_013467963.1	6.4	9.8	6.9	uncharacterized LOC25483798 [*Medicago truncatula* (barrel medic)]
MS.gene07287	XP_003591401.1	8.8	11.2	10.5	calvin cycle protein CP12-2, chloroplastic [*Medicago truncatula* (barrel medic)]
MS.gene24098	PNY14915.1	5.1	5.7	6.2	thioredoxin-like protein CDSP32 chloroplastic-like [*Trifolium pratense*]
MS.gene24746	RHN68722.1	6.5	4.9	5.3	hypothetical protein MtrunA17_Chr3g0116951[*Medicago truncatula*]
MS.gene36621	XP_003627058.1	NA	4.7	4.5	stem 28 kDa glycoprotein [*Medicago truncatula* (barrel medic)]
MS.gene39381	RHN38725.1	NA	6.6	6.5	putative nucleoporin protein Ndc1-Nup [*Medicago truncatula*]
MS.gene63155	RHN41150.1	NA	7.3	4.2	putative protein kinase RLK-Pelle-LRR-XII-1 family [*Medicago truncatula*]
MS.gene81767	XP_013467963.1	NA	4.8	3.6	uncharacterized LOC25483798 [*Medicago truncatula* (barrel medic)]
MS.gene99197	AIP98334.1	4.6	4.8	4.7	ZEP [*Medicago sativa*]

^a^Nr ID is the protein accession number in NCBI non redundant protein database

^b^log2FC stands for log Fold Change, where it is log base 2

**Table 5:** List of 18 salt responsive candidate genes highly expressed in root tissue of salt tolerant alfalfa cultivar ‘Halo’

Gene ID	Nr ID^a^	log_2_FC^b^ (Root)			Putative function
		0h	3h	27h	
MS.gene002389	XP_003593475.1	7.4	8.0	7.9	secretory carrier-associated membrane protein [*Medicago truncatula* (barrel medic)]
MS.gene011517	XP_003608741.1	8.1	8.6	8.6	14 kDa proline-rich protein DC2.15 [*Medicago truncatula* (barrel medic)]
MS.gene013923	XP_003624859.1	6.5	6.6	6.6	histone-lysine N-methyltransferase, H3 lysine-9 specific SUVH1 [*Medicago truncatula* (barrel medic)]
MS.gene023013	XP_013448530.1	8.0	6.0	6.2	peptidyl-prolyl cis-trans isomerase FKBP62 [*Medicago truncatula* (barrel medic)]
MS.gene02427	AFK40071.1	6.9	5.7	5.5	soluble inorganic pyrophosphatase PPA1 [*Medicago truncatula* (barrel medic)]
MS.gene029223	XP_003592714.1	5.9	6.7	6.1	E3 ubiquitin ligase BIG BROTHER-related [*Medicago truncatula* (barrel medic)]
MS.gene044457	XP_013458006.1	7.3	6.4	7.0	uncharacterized LOC25493896 [*Medicago truncatula* (barrel medic)]
MS.gene049130	RDX70942.1	9.2	10.0	8.5	Aldehyde dehydrogenase family 2 member C4, partial [*Mucuna pruriens*]
MS.gene049840	XP_024638826.1	7.4	7.2	8.1	uncharacterized LOC11406476 [*Medicago truncatula* (barrel medic)]
MS.gene056386	XP_013456308.1	8.2	8.3	6.7	fructokinase-2 [*Medicago truncatula* (barrel medic)]
MS.gene058673	PNX87529.1	7.9	5.5	9.5	heavy-metal-associated domain-containing protein [Trifolium pratense]
MS.gene070486	XP_003616935.1	5.7	5.5	7.9	phosphatidylglycerol/phosphatidylinositol transfer protein [*Medicago truncatula* (barrel medic)]
MS.gene073760	XP_013468212.1	7.1	7.8	6.9	probable E3 ubiquitin-protein ligase LOG2 [*Medicago truncatula* (barrel medic)]
MS.gene43277	XP_003608928.1	6.9	7.7	7.4	betaine aldehyde dehydrogenase 1, chloroplastic [*Medicago truncatula* (barrel medic)]
MS.gene46459	XP_013467706.1	8.6	9.5	7.7	uncharacterized LOC25483559 [*Medicago truncatula* (barrel medic)]
MS.gene61130	XP_004487038.1	NA	4.4	4.1	60S ribosomal protein L23a-2-like [ *Cicer arietinum* (chickpea)]
MS.gene67829	XP_013454067.1	9.5	8.7	9.1	ribosomal RNA small subunit methyltransferase nep-1 [*Medicago truncatula* (barrel medic)]
MS.gene95536	XP_013469288.1	6.7	7.4	7.0	acyl-CoA-binding domain-containing protein 6 [*Medicago truncatula* (barrel medic)]

^a^Nr ID is the protein accession number in NCBI non redundant protein database

^b^log2FC stands for log Fold Change, where it is log base 2

In addition, there were also genes consistently expressed under salt stress in leaf (Additional file 1: Table S1) and root (Additional file 1: Table S2) tissues of ‘Vernal’. In ‘Vernal’, there were 21 (17 in leaf; 4 in root) genes consistently expressed at all three time points and 9 (6 in leaf; 3 in root) genes consistently expressed at both 3 h and 27 h of salt stress.

### Identification of single nucleotide polymorphisms (SNPs)

**Fig. 5:**
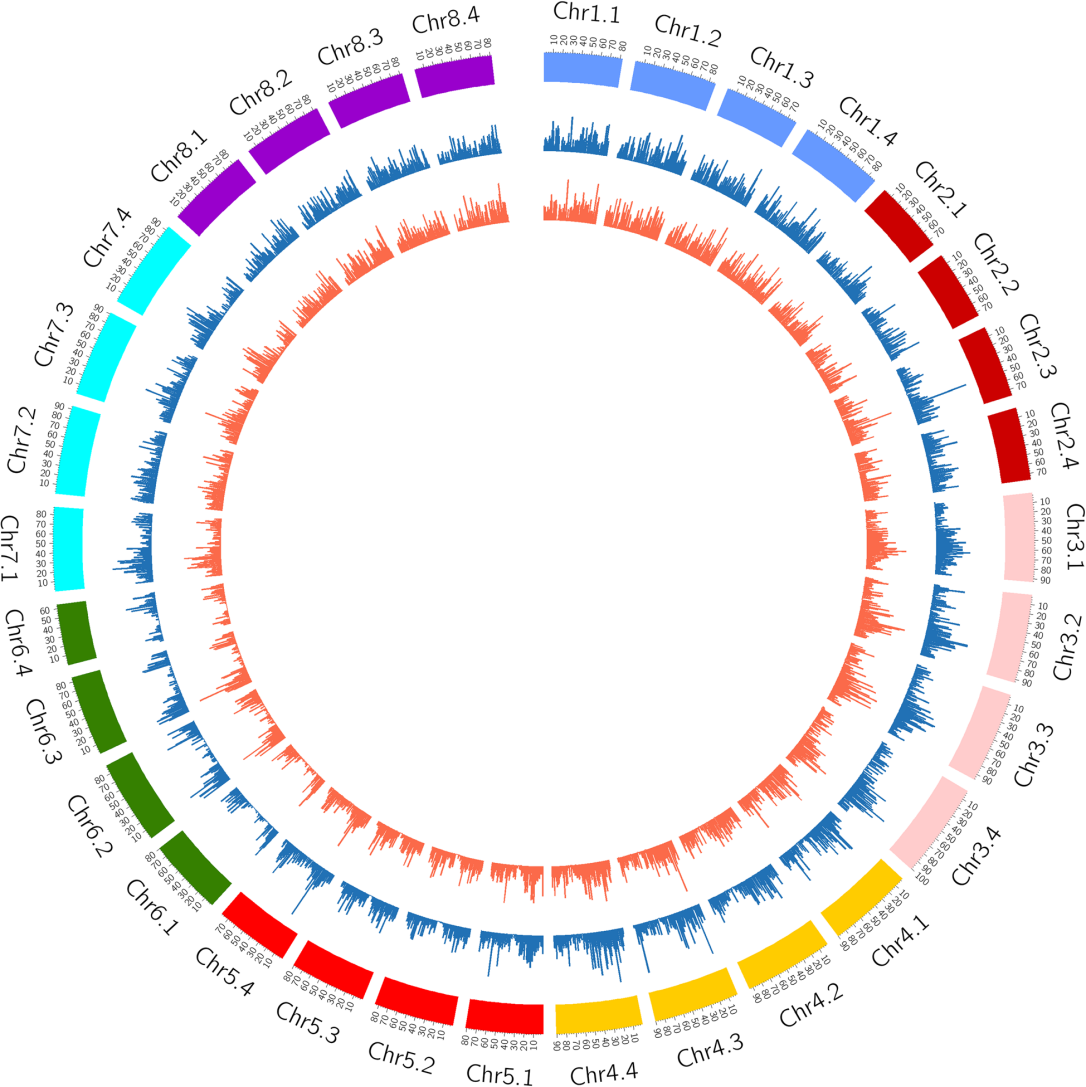
Distribution of SNPs identified over 32 allelic chromosomes of alfalfa (*Medicago sativa* L.) is represented with the Circos diagram. Histogram (1–70) showing distribution of SNPs per Mb bins across genome of alfalfa. The 8 alfalfa chromosomes (Chr1-8) are shown on outermost circle, middle (blue) and innermost (orange) circles represent SNPs distribution of salt intolerant 'Vernal' and salt tolerant 'Halo' alfalfa

The relative distribution of identified SNPs over alfalfa chromosome are presented in Fig. 5. A total of 74,705 SNPs were identified in this study, among which 37,527 were from 'Halo' and 37,178 were from 'Vernal'. Minimum number of SNPs were found in Chr6.4 while maximum number of SNPs were detected in Chr4.4 (Fig. 5).

## Discussion

This study generated a unique set of differentially expressed genes associated with salt tolerance in alfalfa. This finding is not only significant for understanding the temporal expression of genes conditioning salt tolerance in alfalfa, but also can be used to characterize alfalfa breeding material and develop molecular markers for salt tolerance selection. First, 84.8% of sequence reads were mapped onto the alfalfa reference genome. Secondly, 237 DEGs in leaf and 295 DEGs in root tissues were identified between the two alfalfa cultivars. Third, this study was able to determine candidate genes consistently expressed under short-term and long-term salt stress in the salt tolerant cultivar. Fourth, this study found 74,705 SNPs which are valuable marker for future alfalfa breeding for salt tolerance. Fifth, we found ‘Halo’ under salt stress maintained 5 five times more DEGs in the root than ‘Vernal’. Finally, this study found seven candidate genes for salt tolerance (MS.gene32989, MS.gene065734, MS.gene24746, MS.gene81767, MS.gene044457, MS.gene049840, and MS.gene46459) with unknown functions, suggesting a need for further research to understand their role in salt tolerance.

Due to polyploidy and its out-crossing nature, alfalfa has encountered many challenges in genomic studies [26] as compared to self-pollinated crops such as wheat [27] and soybean (*Glycine max*) [28]. Thus, this transcriptomic study had considered several factors to overcome certain technical difficulties. First, identical clones were sampled at different time points from different alfalfa tissues. Unlike previous alfalfa transcriptomic studies, two technical replicates for each treatment were included to minimize technical errors. Furthermore, this study focused on both leaf and root tissues of alfalfa cultivars to capture tissue specific gene expression. These considerations seemed to be effective in capturing about 381 million high quality reads, which likely represents most of the genome of *M. sativa*. The raw reads showed a high percentage of mapping with the reference genome. These outputs should have enhanced our detection of DEGs. For example, both alfalfa cultivars showed a similar trend in the number of DEGs in leaf tissue with the increase of salt exposure time. In this study, we also selected three different time points (0 h, 3 h, and 27 h) to capture gene activation under short- and long- term salt stress. It has been established that salt responsive defense response is activated within 24 h of stress [29].

One of the main differences between the two cultivars was the number of DEGs in roots. In the root of salt tolerant alfalfa, the number of DEGs was similar between 3 h and 27 h of salt stress, but a sharp decrease was observed between 3 h and 27 h in the intolerant cultivar ‘Vernal’. We speculate that such earlier activation of salt responsive genes and maintenance of a large number of DEGs might be a key characteristic for salt tolerance in alfalfa, suggesting alfalfa tolerance is associated with upregulation of key genes from short term salt stress. About 60% of DEGs were assigned to GO categories, while KEGG pathways for less than 30% DEGs were identified in this study. The DEGs were mainly involved in metabolic pathways as revealed by KEGG pathway analysis. Although certain pathways involved in salt tolerance may be conserved in plant species such as in halophytes, there was still variation among plant species, cultivars, and tissues [5]. This study demonstrated that transcriptional variation in adaptation to salt stress exists not only among the alfalfa cultivars but also between the different tissues. ‘Ion binding’ (GO:0043167) was significantly enriched in both leaf and root tissues of ‘Halo’, but not in ‘Vernal’ under salt stress. This suggested that the genes responsible for ‘ion binding’ should be unique for salt tolerance of ‘Halo’ alfalfa. Therefore, the tissue- and genotype-specific salt responsive genes might be useful in identification of salt tolerant genotypes in the future.

Among 13 candidate genes expressed in leaf and root tissues of ‘Halo’ under salt stress (Table 3) in this study, two genes (MS.gene013222 and MS.gene52595) are responsible for transmembrane protein function. These transmembrane proteins control gateways and selective transport of salt ions to facilitate salt tolerance in plants. Likewise, MS.gene013211, a homologous gene to ribonuclease TUDOR1, is involved in stress adaptation and highly expressed in leaf and root tissues of ‘Halo’ in our study [30]. MS.gene93979, a homologous gene to NF-X1-type zinc finger protein, is part of mechanisms that regulate growth under salt stress and was highly expressed in leaf and root tissues of ‘Halo’ in our study [31]. In addition, MS.gene029202 (E3 ubiquitin-protein ligase CIP8), MS.gene029203 (F-box/LRR-repeat protein 4), MS.gene36780 and MS.gene36960 (elongation factor 1-alpha) were highly expressed in leaf and root tissues of salt tolerant alfalfa in our study. These genes are involved in regulation of a number of biological processes including biotic and abiotic stress tolerances [32–34]. For example, MS.gene049294, which is a homologous gene of O-methyltransferase, was found to improve salt tolerance in transgenic Arabidopsis [35]. MS.gene01091, a homologous gene to the T-complex protein 1 subunit gamma, showed high expression in both root and leaf tissue and is involved in intracellular assembly and folding of various proteins [36]. MS.gene029200, a homologous gene to replication factor A protein, was highly expressed in both leaf and root tissues of ‘Halo’ in our study, which might play a role in binding, replication, repair, and recombination of DNA under stress conditions [37].

In this study, we found 15 and 18 candidate genes specific to leaf and root tissues of salt tolerance ‘Halo’ alfalfa (Tables 4, 5). In leaf tissue, nine genes showed consistent expression under salt stress, while six of them were expressed at all three time points. In our study, salt tolerant alfalfa showed an increased expression of MS.gene024018 and MS.gene24098 with putative functions of chloroplastic glutaredoxin and thioredoxin-like protein CDSP32, respectively. The two genes (MS.gene024018, MS.gene24098) were found to be important for defense against protein oxidative damage in other studies [38, 39]. This is important because salt stress results in the formation of reactive oxygen species, which damage protein, membrane lipids, and nucleic acids [40]. MS.gene63155, a homologous gene to receptor-like kinases (RLKs), are a family of transmembrane proteins, showed lowered expression with time under salt stress. This gene is involved in plant growth as well as stress response [41]. Two nucleoporin proteins (MS.gene037960 and MS.gene39381) were expressed consistently under salt stress in leaf tissue of ‘Halo’. These proteins connect cytoplasm and nucleoplasm, and are involved in abiotic stress tolerance [42]. MS.gene038586 is a homologous gene to kinesin super family proteins which plays a significant role in intracellular transport and are critical for cellular functioning and survival [43]. MS.gene029206, a homologous gene to FAD synthetase 1, is a co-factor for various enzymes that participate in numerous metabolic processes like photosynthesis, electron transport, fatty acid oxidation and biosynthesis of secondary metabolites [44]. MS.gene36621, a homologous gene to stem 28 kDa glycoprotein, which is known as a vegetative storage protein, was highly expressed under salt stress in our study. This protein plays a certain role as a somatic storage protein during early seedling development [45]. Salt tolerant alfalfa showed a high expression of MS.gene07287 in leaf, a homologous gene to calvin cycle protein CP12-2. This gene is involved in photosynthesis and improved plant growth [46]. The Calvin–Benson cycle is the primary pathway of carbon fixation, producing carbon compounds. CP12 facilitates the formation of a complex between glyceraldehyde-3-phosphate dehydrogenase and phosphoribulokinase, thereby increasing the photosynthetic capacity of the plant [46]. MS.gene99197, a homologous gene to zeaxanthin epoxidase (ZEP), was highly expressed at all three time points in leaf tissue of ‘Halo’. It is an important enzyme in ABA biosynthesis and plays an important role in osmotic tolerance [47].

In root tissue, one (MS.gene61130) of the 18 genes detected was consistently expressed under salt stress, while the rest were expressed at all three time points in our study (Table 5). MS.gene002389, a homologous gene to secretory carrier-associated membrane proteins, is involved in membrane trafficking and found to influence accumulation of secondary cell wall components in *Poplus* [48]. MS.gene011517, a homologous gene to 14-kDa proline-rich protein DC2.15, is involved in cell wall modification and organization [49]. The plant cell wall is not only a physical barrier between the plant and the environment but also is a responsive part of the plant to biotic and abiotic stresses. The finding of tissue specific salt tolerant candidate genes responsible for the plant cell wall is promising and underlines the need for further research on its role in response to salt stress. In addition, salt stress causes lipid peroxidation, resulting in damage of membrane lipids and eventual cell leakage. This study showed salt tolerant alfalfa had an increased expression of MS.gene049130, a homologous gene to aldehyde dehydrogenase, responsible for oxidation of aldehydes produced during lipid peroxidation thereby detoxifying cells [50]. MS.gene95536 is a homologous gene to acyl-CoA-binding domain-containing protein 6, which is associated with phospholipid metabolism. This gene also was shown to play a role in the freezing tolerance of *Arabidopsis* [51]. MS.gene070486, a homologous gene to phosphatidylinositol transfer proteins, plays an important role in signal transduction and facilitates lipid transfer between membranes [52]. MS.gene056386, a homologous gene to fructokinases, are important enzymes catalyzing fructose phosphorylation and are involved in plant growth and development [53]. MS.gene058673, a homologous gene to heavy-metal-associated domain-containing protein conferring tolerance to abiotic stress [54]. MS.gene073760, a homologous gene to probable E3 ubiquitin-protein ligase LOG2, which induces amino acid secretion. This is the main form of organic nitrogen in the plant [55]. MS.gene02427, a homologous gene to soluble inorganic pyrophosphatase, is tightly linked with carbohydrate metabolism. It plays an important role in stress adaptive responses [56]. Carbohydrate metabolism produces soluble carbohydrates that are important for salt tolerance because of its osmotic adjustment function in the root.

## Conclusion

Our study generated a unique set of DEGs for alfalfa salt tolerance studies and breeding efforts. The information is useful for better understanding of temporal expression of genes in response to salt stress. Furthermore, GO annotation and KEGG pathway analysis of the DEGs provided insights to the different molecular and biological processes between salt tolerant and intolerant alfalfa cultivars. In particular, ‘ion binding activity’ was found as a key molecular activity specific to salt tolerant alfalfa cultivar ‘Halo’. Based on this finding, salt tolerance in alfalfa appears to be associated with consistent expression of genes for selective transport of salt ions and compounds, increasing photosynthetic capacity as well as carbohydrate metabolism, enhancing defense against oxidative damage, modification of root cell wall and protection against lipid peroxidation. The SNPs discovered in this study will be valuable for molecular marker-assisted breeding for the development of salt tolerant alfalfa.

## Methods

### Plant material and salt treatment

Two alfalfa cultivars, ‘Halo’ (obtained from Agriculture and Agri-Food Canada, Swift Current Research and Development Centre) and ‘Vernal’ (sourced from Dr. Biligetu’s lab, Crop Development Centre, University of Saskatchewan) were chosen for the study. Cultivar ‘Halo’ was selected for improved salinity tolerance for germination, seedling growth, and mature plant regrowth at 100 mM NaCl in the greenhouse conditions [57], and cultivar ‘Vernal’ was considered as a salinity intolerant cultivar [58, 59]. Four genotypes (biological replicates) of each cultivar were grown from seeds in the College of Agriculture and Bioresources greenhouse at the University of Saskatchewan (45 Innovation Blvd., Saskatoon, SK) for 12 weeks. Six identical clones of each biological replicate were produced by stem cuttings. Salt stress of 120 mM NaCl approximately corresponding to 12 dS m^− 1^ electrical conductivity was applied on 4 week old seedlings. Salt stress of 12 dS m^− 1^ was selected from our earlier greenhouse study where alfalfa was grown at various gradients of salt stress and alfalfa cultivars showed variation in response to salt stress at 12 dS m^− 1^, with increase in salt stress from 12 dS m^− 1^ all alfalfa cultivars showed very high mortality (Bhattarai et al., unpublished). Leaf and root samples were collected immediately before salt treatment (control, 0 h), and at 3 h and 27 h of salt treatments. The samples were immediately frozen in liquid nitrogen and then stored at − 80 °C for 2 weeks until total RNA extraction carried out.

### Tissue sample and RNA isolation

About 100 mg of tissue samples were disrupted using TissueLyser II and total RNA was extracted with RLT buffer using the Qiagen RNeasy Plant Mini Kit (Qiagen Inc., Mississauga, ON, Canada) according to the manufacturer’s protocol. DNase treatment was performed using the Ambion DNA-free DNase treatment and removal reagents (Life Technologies, Carlsbad, CA, USA) to remove contaminant genomic DNA from the isolated total RNA. Nanodrop 2000 (Thermo Fisher Scientific, Wilmington, DE, USA) was used to measure the total RNA concentration. RNA integrity number was evaluated for 12 samples using RNA 6000 Nano labchip on 2100 Agilent Bioanalyzer (Agilent Technologies, Waldbronn, Germany) (Additional file 1: Table S3; Additional file 2: Fig. S1).

### Library preparation and sequencing

Poly (A) RNA was purified from total RNA using Magnosphere MS150 OligodT beads according to the manufacturer’s protocol. The RNA samples were subsequently used in cDNA library preparation. Two cDNA libraries were prepared using Lexogen’s SENSE mRNA-Seq Library Prep Kit V2 (Lexogen, Vienna, Austria). To minimize technical errors, two technical replicates of each treatment were divided into two cDNA libraries. The technical replicates represented two clones of the same genotype (biological replicate) by separately extracting RNA. Thus, 96 samples (2 cultivars × 2 tissue types × 3 time points × 4 biological replicates × 2 technical replicates) were collected for the study. The cDNA libraries were sequenced using the Illumina HiSeq v4 system at the National Research Council of Canada, Saskatoon, Canada. Raw reads were deposited in the National Center for Biotechnology Information (NCBI) and received BioProject ID PRJNA657410.

### Reference-based mapping, differential gene expression analysis and annotation

The quality of the raw sequence was assessed using the FastQC software [60]. The raw reads were cleaned by removing adapters and low-quality sequences using Trimmomatic v.0.36 based on the default setting of paired-end mode, phred 33 and threads 6 [61]. The trimmed high-quality reads of samples from the two technical replicates were merged and mapped with the alfalfa reference genome (10.6084/m9.figshare.12327602.v3) [62, 63] using STAR (v2.6.1a) [64] with “quantMode” as “GeneCounts”. The obtained “ReadsPerGene” of each sample were extracted as count matrix and the differentially expressed genes were analyzed using DeSeq2 package [65] where data were normalized by the median of the ratios. The threshold of padj < 0.001 and the Log fold change (Log2FC) > 2 were used to determine the significance of gene expression differences. The functional annotation of the DEGs were also extracted via searches of NR databases as available in “query.blastp.db.out” and gene ontologies were obtained via searches of the GO databases as available in “Msa.GO.list.up” likewise Kyoto Encyclopedia of Genes and Genomes (KEGG) Ortholog (KO) were obtained via search of KO databases as available in “query.ko” from Zeng [63]. Gene ontology analysis of the DEGs was done for biological process, cellular components, and molecular function by AgriGO v2.0 software [66]. Venn diagrams were produced using the Venny tool [67].

### Identification of single nucleotide polymorphisms (SNPs)

SNPs calling was done using freebayes software using the bam file generated in the mapping process where at least 5 supporting observations were required to be consider a variant [68]. To visualize the relative distribution of SNPs over chromosomes, Circos tool was used [69].

## Supplementary Information

**Additional file 1 **: **Table S1.** List of 237 differentially expressed genes in leaf tissue at the control (0 h), 3 h, and 27 h of salt stress between salt tolerant ‘Halo’ and salt intolerant ‘Vernal’ cultivars of alfalfa. **Table S2.** List of 295 differentially expressed genes in root tissue at the control (0 h), 3 h, and 27 h of salt stress between salt tolerant ‘Halo’ and salt intolerant ‘Vernal’ cultivars of alfalfa. **Table S3.** RNA quality of 12 RNA samples determined with 2100 Agilent Bioanalyzer.

**Additional file 2 **: **Fig. S1.** Electropherogram of 12 RNA samples.

## Data Availability

Raw reads have been deposited in the National Center for Biotechnology Information (NCBI) and received BioProject ID PRJNA657410. The data will be accessible with the following link: “https://www.ncbi.nlm.nih.gov/bioproject/PRJNA657410”.

## Abbreviations

ABA: Abscisic acid
AVP1: Arabidopsis type I proton-pumping pyrophosphatase
DEGs: Differentially expressed genes
dS m^− 1^: DeciSiemens per metre
DREB: Dehydration-responsive element binding protein
NHX: Na^+^/H^+^ antiporter
CBRLK: Calcium/calmodulin-binding receptor-like kinase
ZFP: Zinc finger protein
APX: Ascorbate peroxidase
NDPK2: Nucleoside diphosphate kinase 2
codA: Choline oxidase
RLK: Receptor-like kinase
FAD: Flavin adenine dinucleotide
ZEP: Zeaxanthin epoxidase

## References

[1] Blondon F, Marie D, Brown S, Kondorosi A (1994). Genome size and base composition in *Medicago* siativa and *M. truncatula* species. Genome..

[2] Maas EV, Hoffman GJ (1977). Crop salt tolerance-current assessment. J Irrig Drain Div.

[3] Johnson DW, Smith SE, Dobrenz AK (1992). Selection for increased forage yield in alfalfa at different NaCl levels. Euphytica..

[4] Gregorio GB, Senadhira D (1993). Genetic analysis of salinity tolerance in rice (*Oryza sativa* L.). Theoret Appl Genet.

[5] Munns R, Tester M. Mechanisms of salinity tolerance. Annu Rev Plant Biol. 2008;59(1):651–81. 10.1146/annurev.arplant.59.032607.092911. 10.1146/annurev.arplant.59.032607.092911 18444910

[6] Postnikova OA, Shao J, Nemchinov LG (2013). Analysis of the alfalfa root transcriptome in response to salinity stress. Plant Cell Physiol.

[7] Luo D, Zhou Q, Wu YG, Chai XT, Liu WX, Wang YR, Yang QC, Wang ZY, Liu ZP (2019). Full length transcript sequencing and comparative transcriptomic analysis to evaluate the contribution of osmotic and ionic stress components towards salinity tolerance in the roots of cultivated alfalfa (*Medicago sativa* L.). BMC Plant Biol.

[8] Lei Y, Xu Y, Hettenhausen C, Lu C, Shen G, Zhang C, Li J, Song J, Lin H, Wu J (2018). Comparative analysis of alfalfa (*Medicago sativa* L.) leaf transcriptomes reveals genotype-specific salt tolerance mechanisms. BMC Plant Biol.

[9] Gruber M, Xia J, Yu M, Steppuhn H, Wall K, Messer D, Sharpe A, Acharya S, Wishart D, Johnson D, Miller D, Taheri A (2017). Transcript analysis in two alfalfa salt tolerance selected breeding populations relative to a non-tolerant population. Genome..

[10] Winicov I (2000). Alfin1 transcription factor overexpression enhances plant root growth under normal and saline conditions and improves salt tolerance in alfalfa. Planta..

[11] Bao AK, Wang SM, Wu GQ, Xi JJ, Zhang JL, Wang CM (2009). Overexpression of the Arabidopsis *H+-PPase* enhanced resistance to salt and drought stress in transgenic alfalfa (*Medicago sativa* L.). Plant Sci.

[12] Jin T, Chang Q, Li W, Yin D, Li Z, Wang D, Liu B, Liu L (2010). Stress-inducible expression of *GmDREB1* conferred salt tolerance in transgenic alfalfa. Plant Cell Tissue Organ Cult.

[13] Li W, Wang D, Jin T, Chang Q, Yin D, Xu S, Liu B, Liu L (2011). The vacuolar Na+/H+ antiporter gene *SsNHX1from* the halophyte Salsola soda confers salt tolerance in transgenic alfalfa (*Medicago sativa* L.). Plant Mol Biol Rep.

[14] Zhang YM, Liu ZH, Wen ZY, Zhang HM, Yang F, Guo XL (2012). The vacuolar Na+/H+ antiport gene *TaNHX2 confers* salt tolerance on transgenic alfalfa (*Medicago sativa* L.). Funct Plant Biol.

[15] Bai X, Liu J, Tang L, Cai H, Chen M, Ji W, Liu Y, Zhu Y (2013). Overexpression of *GsCBRLK* from *Glycine soja* enhances tolerance to salt stress in transgenic alfalfa (*Medicago sativa*). Funct Plant Biol.

[16] Tang L, Cai H, Ji W, Luo X, Wang Z, Wu J, Wang X, Cui L, Wang Y, Zhu Y, Bai X (2013). Overexpression of *GsZFP1enhances* salt and drought tolerance in transgenic alfalfa (*Medicago sativa* L.). Plant Physiol Biochem.

[17] Zhang Q, Ma C, Xue X, Xu M, Li J, Wu JX (2014). Overexpression of a cytosolic ascorbate peroxidase gene, *OsAPX2*, increases salt tolerance in transgenic alfalfa. J Integr Agric.

[18] Zhang LQ, Niu YD, Huridu H, Hao JF, Qi Z, Hasi A (2014). *Salicornia europaea* L. Na+/H+ antiporter gene improves salt tolerance in transgenic alfalfa (*Medicago sativa* L.). Genet Mol Res.

[19] Wang Z, Li H, Ke Q, Jeong JC, Lee HS, Xu B, Deng XP, Lim YP, Kwak SS (2014). Transgenic alfalfa plants expressing *AtNDPK2* exhibit increased growth and tolerance to abiotic stresses. Plant Physiol Biochem.

[20] Li H, Wang Z, Ke Q, Ji CY, Jeong JC, Lee H, Lim YP, Xu B, Deng XP, Kwak SS (2014). Overexpression of *codA* gene confers enhanced tolerance to abiotic stresses in alfalfa. Plant Physiol Biochem.

[21] Tang L, Cai H, Zhai H, Luo X, Wang Z, Cui L, Bai X (2014). Overexpression of Glycine soja *WRKY20* enhances both drought and salt tolerance in transgenic alfalfa (*Medicago sativa* L.). Plant Cell Tissue Organ Cult.

[22] Ramagopal S (1987). Salinity stress induced tissue-specific proteins in barley seedling. Plant Physiol.

[23] Ramagopal S (1987). Differential mRNA transcription during salinity stress in barley. Proc Natl Acad Sci U S A.

[24] Kumar S, Beena AS, Awana M, Singh A (2017). Salt-induced tissue-specific cytosine methylation downregulates expression of HKT genes in contrasting wheat (*Triticum aestivum* L.) genotypes. DNA Cell Biol.

[25] Villarino GH, Hu Q, Scanlon MJ, Mueller L, Bombarely A, Mattson NS (2017). Dissecting tissue-specific transcriptomic responses from leaf and roots under salt stress in *Petunia hybrida* Mitchell. Genes..

[26] Liu Z, Chen T, Ma L, Zhao Z, Zhao PX, Nan Z, Wang Y (2013). Global transcriptome sequencing using the Illumina platform and the development of EST-SSR markers in autotetraploid alfalfa. PLoS One.

[27] Feldman M, Levy AA (2005). Allopolyploidy – a shaping force in the evolution of wheat genomes. Cytogenet Genome Res.

[28] Gill N, Findley S, Walling JG, Hans C, Ma J, Doyle J, Stacey G, Jackson SA (2009). Molecular and chromosomal evidence for allopolyploidy in soybean. Plant Physiol.

[29] Hernandez JA, Almansa MS (2002). Short-term effects of salt stress on antioxidant systems and leaf water relations of pea leaves. Physiol Plant.

[30] Yan C, Yan Z, Wang Y, Yan X, Han Y (2014). Tudor-SN, a component of stress granules, regulates growth under salt stress by modulating GA20ox3 mRNA levels in Arabidopsis. J Exp Bot.

[31] Lisso J, Altmann T, Müssig C (2006). The *AtNFXL1* gene encodes a NF-X1 type zinc finger protein required for growth under salt stress. FEBS Lett.

[32] Mazzucotelli E, Belloni S, Marone D, De Leonardis A, Guerra D, Di Fonzo N, Cattivelli L, Mastrangelo A (2006). The e3 ubiquitin ligase gene family in plants: regulation by degradation. Curr Genomics.

[33] Song JB, Wang YX, Li HB, Li BW, Zhou ZS, Gao S, Yang ZM (2015). The F-box family genes as key elements in response to salt, heavy metal and drought stresses in *Medicago truncatula*. Funct Integr Genomics.

[34] Gao Y, Ma J, Zheng JC, Chen J, Chen M, Zhou YB, Fu JD, Xu ZS, Ma YZ (2019). The elongation factor GmEF4 is involved in the response to drought and salt tolerance in soybean. Int J Mol Sci.

[35] Niron H, Türet M (2020). A putative common bean chalcone o-methyltransferase improves salt tolerance in transgenic *Arabidopsis thaliana*. J Plant Growth Regul.

[36] Bhaskar KN, Goyal N (2012). Cloning, characterization and sub-cellular localization of gamma subunit of T-complex protein-1 (chaperonin) from *Leishmania donovani*. Biochem Biophys Res Commun.

[37] Longhese MP, Plevani P, Lucchini G (1994). Replication factor A is required in vivo for DNA replication, repair, and recombination. Mol Cell Biol.

[38] Broin M, Rey P (2003). Potato plants lacking the *CDSP32* plastidic thioredoxin exhibit overoxidation of the BAS1 2-cysteine peroxiredoxin and increased lipid peroxidation in thylakoids under photooxidative stress. Plant Physiol.

[39] Cheng NH, Liu JZ, Brock A, Nelson RS, Hirschi KD (2006). *AtGRXcp*, an Arabidopsis chloroplastic glutaredoxin, is critical for protection against protein oxidative damage. J Biol Chem.

[40] Foyer CH, Noctor G (2005). Oxidant and antioxidant signaling in plants: a re-evaluation of the concept of oxidative stress in a physiological context. Plant Cell Environ.

[41] Shiu SH, Bleecker AB (2003). Expansion of the receptor-like kinase/Pelle gene family and receptor-like proteins in Arabidopsis. Plant Physiol.

[42] Dong CH, Hu X, Tang W, Zheng X, Kim YS, Lee BH, Zhu JK (2006). A putative Arabidopsis nucleoporin, *AtNUP160*, is critical for RNA export and required for plant tolerance to cold stress. Mol Cell Biol.

[43] Hirokawa N, Noda Y (2008). Intracellular transport and kinesin superfamily proteins, KIFs: structure, function, and dynamics. Physiol Rev.

[44] Sandoval FJ, Zhang Y, Roje S (2008). Flavin nucleotide metabolism in plants: monofunctional enzymes synthesize fad in plastids. J Biol Chem.

[45] Mason HS, Guerrero FD, Boyer JS, Mullet JE (1988). Proteins homologous to leaf glycoproteins are abundant in stems of dark-grown soybean seedlings. Analysis of proteins and cDNAs. Plant Mol Biol.

[46] López-Calcagno PE, Abuzaid AO, Lawson T, Raines CA (2017). Arabidopsis CP12 mutants have reduced levels of phosphoribulokinase and impaired function of the Calvin-Benson cycle. J Exp Bot.

[47] Park HY, Seok HY, Park BK, Kim SH, Goh CH, Lee BH, Lee CH, Moon YH (2008). Overexpression of *Arabidopsis ZEP* enhances tolerance to osmotic stress. Biochem Biophys Res Commun.

[48] Obudulu O, Mähler N, Skotare T, Bygdell J, Abreu IN, Ahnlund M, Gandla ML, Petterle A, Moritz T, Hvidsten TR, Jönsson LJ, Wingsle G, Trygg J, Tuominen H (2018). A multi-omics approach reveals function of secretory carrier-associated membrane proteins in wood formation of *Populus* trees. BMC Genomics.

[49] Fan W, Lou HQ, Gong YL, Liu MY, Wang ZQ, Yang JL, Zheng SJ (2014). Identification of early Al-responsive genes in rice bean (*Vigna umbellata*) roots provides new clues to molecular mechanisms of Al toxicity and tolerance. Plant Cell Environ.

[50] Koncitikova R, Vigouroux A, Kopecna M, Andree T, Bartos J, Sebela M, Morera S, Kopecny D (2015). Role and structural characterization of plant aldehyde dehydrogenases from family 2 and family 7. Biochem J.

[51] Chen QF, Xiao S, Chye ML (2008). Arabidopsis *ACBP6* is an acyl-CoA-binding protein associated with phospholipid metabolism. Plant Signal Behav.

[52] Garner K, Hunt AN, Koster G, Somerharju P, Groves E, Li M, Raghu P, Holic R, Cockcroft S (2012). Phosphatidylinositol transfer protein, cytoplasmic 1 (*PITPNC1*) binds and transfers phosphatidic acid. J Biol Chem.

[53] Stein O, Granot D (2018). Plant Fructokinases: evolutionary, developmental, and metabolic aspects in sink tissues. Front Plant Sci.

[54] Sun XH, Yu G, Li JT, Jia P, Zhang JC, Jia CG, Zhang YH, Pan HY (2014). A heavy metal-associated protein (*AcHMA1*) from the halophyte, *Atriplex canescens* (Pursh) Nutt., confers tolerance to iron and other abiotic stresses when expressed in *Saccharomyces cerevisiae*. Int J Mol Sci.

[55] Pratelli R, Guerra DD, Yu S, Wogulis M, Kraft E, Frommer WB, Callis J, Pilot G (2012). The ubiquitin E3 ligase LOSS OF GDU2 is required for GLUTAMINE DUMPER1-induced amino acid secretion in Arabidopsis. Plant Physiol.

[56] Lee SK, Jeon JS (2020). Review: Crucial role of inorganic pyrophosphate in integrating carbon metabolism from sucrose breakdown to starch synthesis in rice endosperm. Plant Sci.

[57] Steppuhn H, Acharya SN, Iwaasa AD, Gruber M, Miller DR (2012). Inherent responses to root-zone salinity in nine alfalfa populations. Can J Plant Sci.

[58] Peel MD, Waldron BL, Jensen KB, Chatterton NJ, Horton H, Dudley LM (2004). Screening for salinity tolerance in alfalfa. Crop Sci.

[59] Rahman MA, Alam I, Kim YG, Ahn NY, Heo SH, Lee DG, Liu G, Lee BH (2015). Screening for salt responsive proteins in two contrasting alfalfa cultivars using a comparative proteome approach. Plant Physiol Biochem.

[60] Schmieder R, Edwards R (2011). Quality control and preprocessing of metagenomic datasets. Bioinformatics..

[61] Bolger AM, Lohse M, Usadel B (2014). Trimmomatic: a flexible trimmer for Illumina sequence data. Bioinformatics..

[62] Chen H, Zeng Y, Yang Y, Huang L, Tang B, Zhang H, Hao F, Liu W, Li Y, Liu Y, Zhang X, Zhang R, Zhang Y, Li Y, Wang K, He H, Wang Z, Fan G, Yang H, Bao A, Shang Z, Chen J, Wang W, Qiu Q (2020). Allele-aware chromosome-level genome assembly and efficient transgene-free genome editing for the autotetraploid cultivated alfalfa. Nat Commun.

[63] Zeng Y (2020). Genome fasta sequence and annotation files. figshare. Dataset.

[64] Dobin A, Davis CA, Schlesinger F, Drenkow J, Zaleski C, Jha S, Batut P, Chaisson M, Gingeras TR (2013). STAR: ultrafast universal RNA-seq aligner. Bioinformatics..

[65] Love MI, Huber W, Anders S (2014). Moderated estimation of fold change and dispersion for RNA-seq data with DESeq2. Genome Biol.

[66] Tian T, Liu Y, Yan H, You Q, Yi X, Du Z, Xu W, Su Z (2017). AgriGO v2.0: a GO analysis toolkit for the agricultural community, 2017 update. Nucleic Acids Res.

[67] Oliveros JC (2007). VENNY. An interactive tool for comparing lists with Venn Diagrams.

[68] Garrison E, Marth G (2012). Haplotype-based variant detection from short-read sequencing.

[69] Krzywinski M, Schein J, Birol I, Connors J, Gascoyne R, Horsman D, Jones SJ, Marra MA (2009). Circos: an information aesthetic for comparative genomics. Genome Res.

