# Supplementary figures and images for: Transcriptomic analysis of differentially expressed genes in leaves and roots of two alfalfa (Medicago sativa L.) cultivars with different salt tolerance
Source: BMC Plant Biol. 2021 Oct 5;21:446. doi: 10.1186/s12870-021-03201-4 (PMC8491396; doi:10.1186/s12870-021-03201-4)

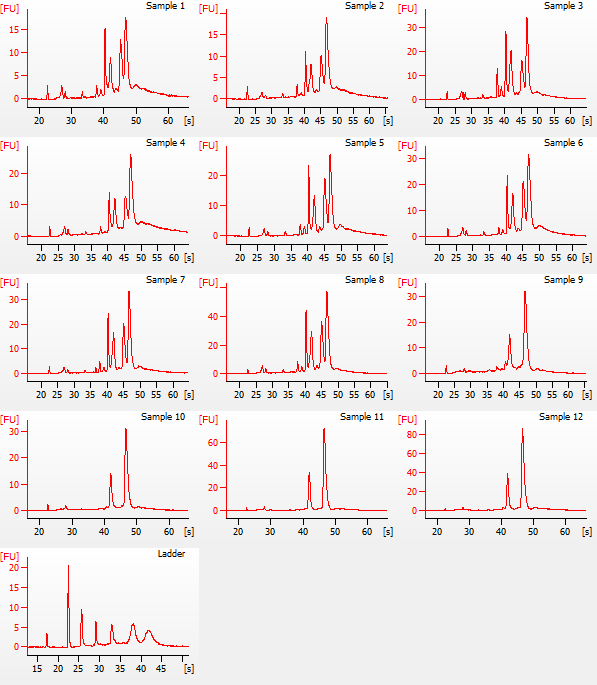

Supplement: Supplementary file 2 — Additional file 2 : Fig. S1. Electropherogram of 12 RNA samples. [file 12870_2021_3201_MOESM2_ESM.tiff]
